# Effects of Alpha-Lytic Therapy Withdrawal on Choroidal Parameters in Intraoperative Floppy Iris Syndrome High-Risk Patients

**DOI:** 10.3390/jcm13247543

**Published:** 2024-12-11

**Authors:** Marco Gioia, Maddalena De Bernardo, Martina De Luca, Nicola Rosa

**Affiliations:** Department of Medicine, Surgery and Dentistry “Scuola Medica Salernitana”, University of Salerno, Via S. Allende, 84081 Baronissi, SA, Italy; marco.gioia@yahoo.it (M.G.); martinadelux@gmail.com (M.D.L.); nrosa@unisa.it (N.R.)

**Keywords:** IFIS, choroid, choroidal thickness, CVI, cataract surgery, cataract, SFCT

## Abstract

**Background/Objectives**: The aim of this study is to investigate the effect on choroidal parameters of drug withdrawal in patients taking α1 adrenergic receptor antagonist (ARA) inhibitors. **Methods**: In total, 32 eyes of 32 patients under alpha-lytic therapy, and 32 eyes of 32 control subjects, both scheduled for cataract surgery in the fellow eye, were included. EDI-OCT was performed in all patients and subfoveal choroidal thickness (SFCT), luminal choroidal area (LCA), stromal choroidal area (SCA), total choroidal area (TCA), and choroidal vascularity index (CVI) during the preoperative visit were compared with data approximately 1 month after alpha-lytic withdrawal. The same assessments were performed in the control group. **Results**: SFCT and LCA were significantly reduced in the patients 1 month after alfa-lytic therapy withdrawal (*p* < 0.05), whereas in the control group, no changes in choroidal parameters were observed. **Conclusions**: The data proved that α1ARA inhibitors affected choroidal vessels, whereas no modification of choroidal stroma was observed.

## 1. Introduction

Cataract surgery becomes very hard in patients with insufficient pupil dilation because surgeons are forced to operate in closed spaces and an increase in complications is observed. Several drugs, such as selective α1 adrenergic receptor antagonist (ARA) inhibitors, could lead to miosis with iris stroma surging and billowing and, even if well-constructed wounds are performed and normal intraocular fluid flows are used, the iris could prolapse through surgical incisions, complicating cataract surgery. This phenomenon is known as intraoperative floppy iris syndrome (IFIS) [1].

IFIS is associated with high rates of intraoperative complications, such as iris prolapse, capsulorhexis tear, iris trauma, anterior chamber hemorrhage, zonula dehiscence, posterior capsule rupture, and vitreous loss, as well as postoperative complications, including intraocular pressure elevation and cystoid macular edema [2,3,4,5].

To avoid the onset of IFIS, selective α1ARA inhibitor withdrawal before surgery has been suggested. Unfortunately, this recommendation is not always effective and, to date, no predictive parameters can help to forecast the appropriate time for surgery.

However, in the past few years, it has been proven that α1ARA inhibitors induce not only iris but also choroidal modifications [6,7]. Furthermore, changes related to α1ARA inhibitor effect on the whole uvea have been shown [8,9,10,11,12], and choroidal thickness (ChT) changes after drug withdrawal have been investigated [13]. Therefore, the aim of this study is to better understand the role of stromal and luminal choroidal involvement in the pathogenesis of this disease to precisely determine the withdrawal time needed to minimize the surgical complications.

## 2. Materials and Methods

### 2.1. Patient Selection

This study was consistent with the Tenets of the Declaration of Helsinki and Institutional Review Board approval was obtained (CECS, Cometico Campania Sud prot. n°16544, 3 October 2017). All participants were informed about the purpose of the study and a written informed consent was acquired.

Patients affected by lens opacities, scheduled for cataract surgery in the Eye Department of the University of Salerno, were recruited in this observational study.

All subjects were divided into two patient groups, one made up of patients on alpha-lytic therapy, to control their urinary symptoms or high blood pressure, and the other a control group, including subjects not on therapy.

Patients affected by corneal leukomas, diabetic retinopathy, hypertensive retinopathy from grade II to IV, age-related macular degeneration, central serous chorioretinopathy, glaucoma, macular hole, optic neuropathy, uveitis, hypertension not controlled by medications, diabetes, autoimmune disorders and ocular or systemic diseases, which could have changed choroidal vasculature, and eyes with an axial length (AL) less than 21 mm or more than 26.5 mm were excluded.

Thirty-two eyes of thirty-to consecutive patients, with a mean age of 75.59 ± 6.57 years, ranging from 58 to 85 years, were appraised, of whom twenty-nine were male patients with a previous diagnosis of benign prostatic hypertrophy (BPH), whose urinary symptoms were under control with alpha-lytic therapy (seven patients took tamsulosin, fifteen alfuzosin, and the other seven silodosin), and two females and one male with arterial hypertension (AH), which was controlled by doxazosin. A control group of 32 patients, fourteen males and eighteen females, with a mean age of 74.75 ± 6.63 years, ranging from 60 to 86 years, and without alpha-lytic therapy, were also enrolled for comparison. No age difference was observed between the groups (*p* = 0.564).

Although a sex difference between the groups was observed, to date, the influence of sex on choroidal parameters is still not very clear and many papers have proven that there was no correlation; therefore, we standardized for biometrical parameters [14,15,16,17,18,19].

In both groups, due to dense lens opacities that could have caused fictitious and inaccurate results, or possible changes induced by the surgery, the fellow eyes were examined, whereas the operated ones were excluded.

### 2.2. Clinical and Instrumental Examination

On the day of the preoperative visit, all patients underwent a complete eye evaluation, including clinical history, Snellen uncorrected and best spectacle corrected visual acuity, anterior segment assessment, intraocular pressure (IOP) measurement, fundus examination, AL measurements with IOLMaster (Carl Zeiss Meditec AG, Jena, Germany; version 5.4.4.0006), and spectral-domain (SD) OCT evaluation using EDI mode in 840 nm (Spectralis; Heidelberg Engineering; Heidelberg, Germany; version 6.0). After urologic or cardiovascular consultation, patients were asked to discontinue alpha-lytic therapy to reduce the IFIS risk, and not to change their lifestyles or other medications.

On the day of the surgical procedure, performed approximately 1 month later (range 28–31 days), the OCT evaluation was repeated utilizing the follow-up mode of the device.

### 2.3. OCT Analysis: Choroidal Parameters

A horizontal 30° linear OCT B-scan passing through the fovea was acquired by an expert examiner not aware of the patients’ distribution, with a scanning angle of 308 and with 36 frames per B-scan. Only EDI-OCT images with a high signal-to-noise ratio (minimum of 20 dB) that permitted sufficient choroidal visualization were included [20]. All examinations were performed between 2:00 p.m. and 3:00 p.m., reducing the bias related to diurnal ChT variations [21].

To obtain comparable results, the image brightness was adjusted. All measurements were taken with the built-in software of the device (Heidelberg Eye Explorer HEYEX; Heidelberg Engineering, Heidelberg, Germany). Subfoveal choroidal thickness (SFCT) was measured as the distance between the Retinal Pigment Epithelium (RPE)–Bruch’s membrane complex and the sclera-choroidal junction under the fovea, and all lines were drawn perpendicularly to the RPE–Bruch’s complex [22,23,24,25,26,27,28,29]. Moreover, total choroidal area (TCA), luminal choroidal area (LCA), stromal choroidal area (SCA), and CVI were evaluated in both groups. All collected and analyzed imaging data were reviewed by an expert in OCT evaluation. Macular B-scan images were exported with a 1:1 pixel ratio and processed with ImageJ 1.52q software (National Institutes of Health, Bethesda, ML, USA), utilizing the set scale tool to convert pixels to microns. TCA, LCA, SCA, and CVI were measured and calculated by an examiner who was blind to the patient’s status, as previously suggested [30,31,32].

**Figure 1:**
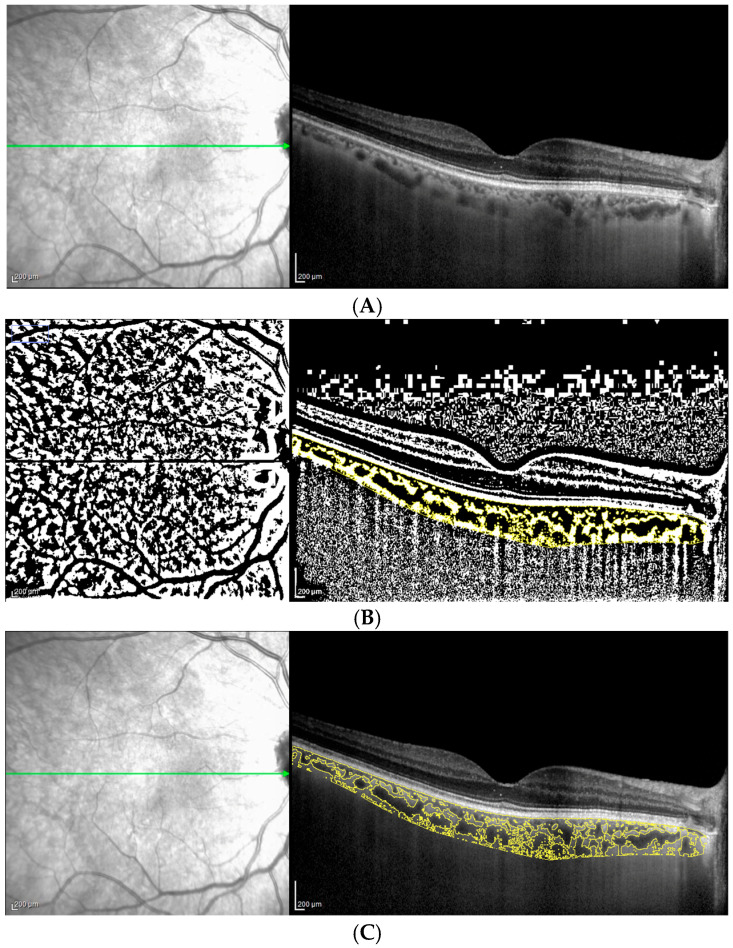
(**A**–**C**) Method of choroidal vascularization parameter computation on ImageJ.

Briefly, the polygon tool was used to select the whole scanned choroidal area in the OCT B-scan. After converting the image into 8 bit, Niblack’s auto-local threshold was applied to binarize the image and demarcate LCA and SCA. TCA was highlighted by applying the color threshold and then added to the region of interest (ROI) manager. TCA and LCA were measured and CVI (defined as the ratio of LCA to TCA) was calculated in each eye (Figure 1A–C). SCA was obtained by subtracting LCA from TCA. To obtain comparable data, the AL assessment for both groups was performed and no statistically significant difference was shown.

### 2.4. Statistical Analysis

The statistical analysis was performed using SPSS Software (IBM SPSS Statistics version 25). The normal distribution of data was assessed with a Kolmogorov–Smirnov test, and all parameters of the patients and controls between the preoperative visit and approximately one month later were compared using a paired two-tailed *t*-test for normally distributed parameters and a Wilcoxon signed-rank test for non-normally distributed data.

The analysis was performed using G*Power3.1 software 7 [33]. A difference between two dependent means (matched pairs) two-tailed *t*-test and a Wilcoxon rank test were computed and results were considered statistically significant for *p* < 0.05.

The input data of the two-tailed *t*-test were the following: α was set at 0.05; 1-β was set at 0.80; and effect size was set as medium at around 0.52. The results were the following: non-centrality parameter δ = 2.941; critical t = 2.039; DF = 31; actual power = 0.81; total sample size = 32.

The input data of the Wilcoxon rank test were the following: α was set at 0.05; 1-β was set at 0.80; and the effect size was set as medium at around 0.53. The results were the following: non-centrality parameter δ = 2.930; critical t = 2.043; DF = 29.558; actual power = 0.81; total sample size = 32.

## 3. Results

The AL measurements in the patients ranged from 22.07 to 26.42 mm (23.61 ± 0.94 mm). In the control group, they ranged from 21.69 to 25.60 mm (23.37 mm ± 0.93 mm), with no significant difference (*p* = 0.996).

**Figure 2:**
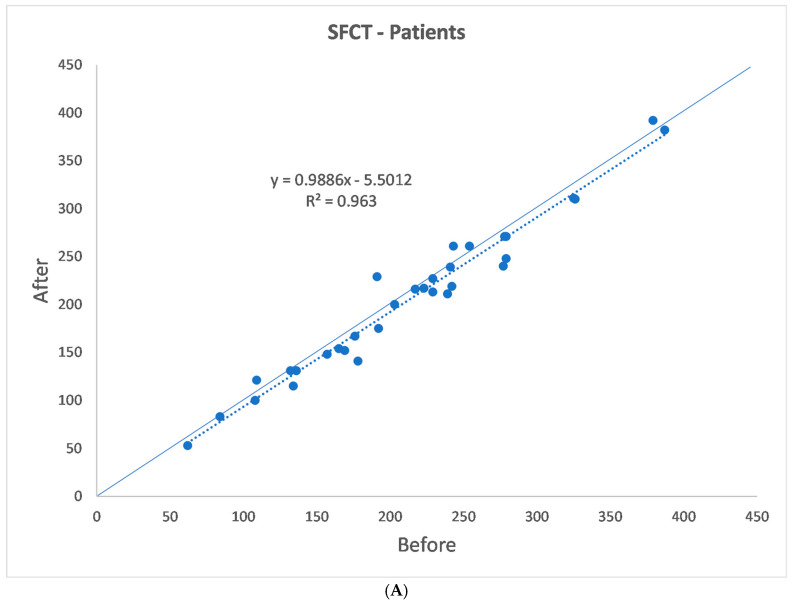

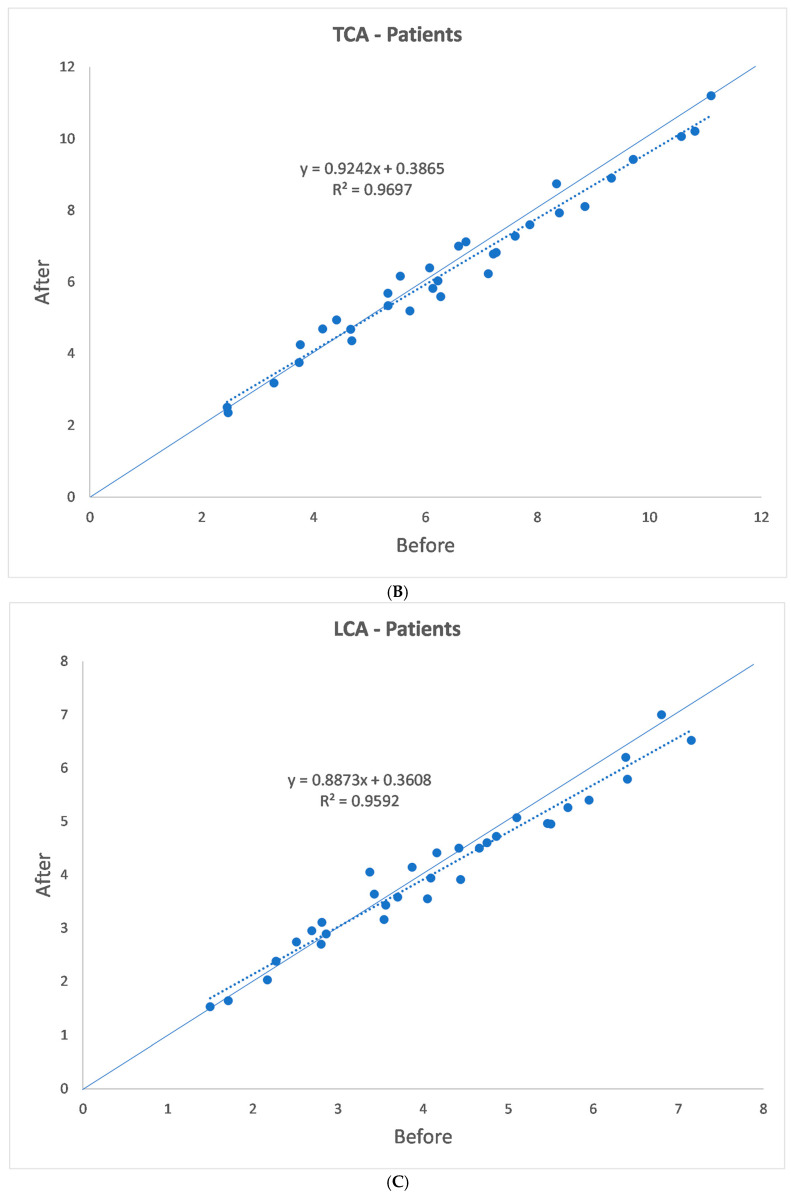

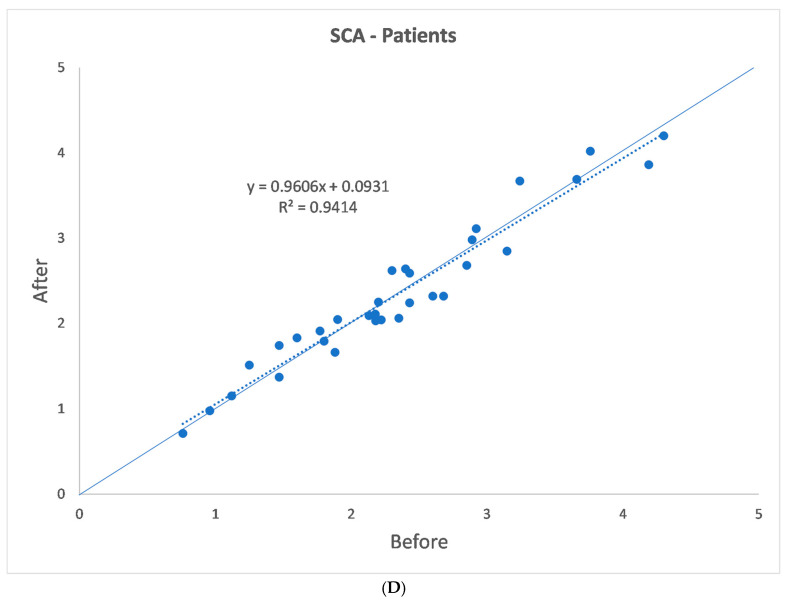
(**A**–**D**) Patients’ choroidal vascularization parameters.

**Figure 3:**
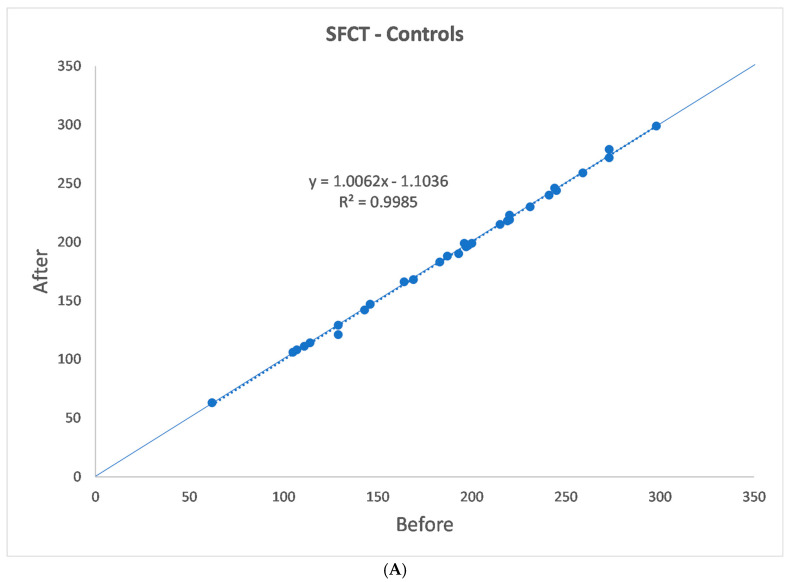

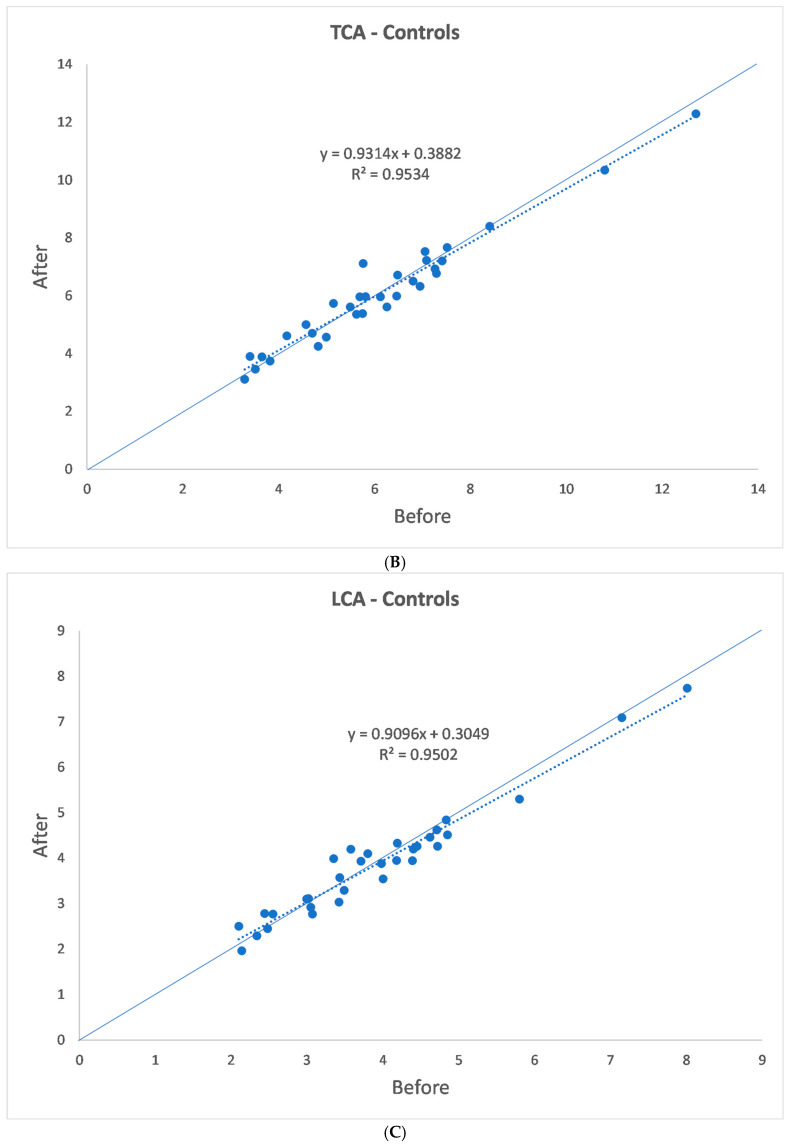

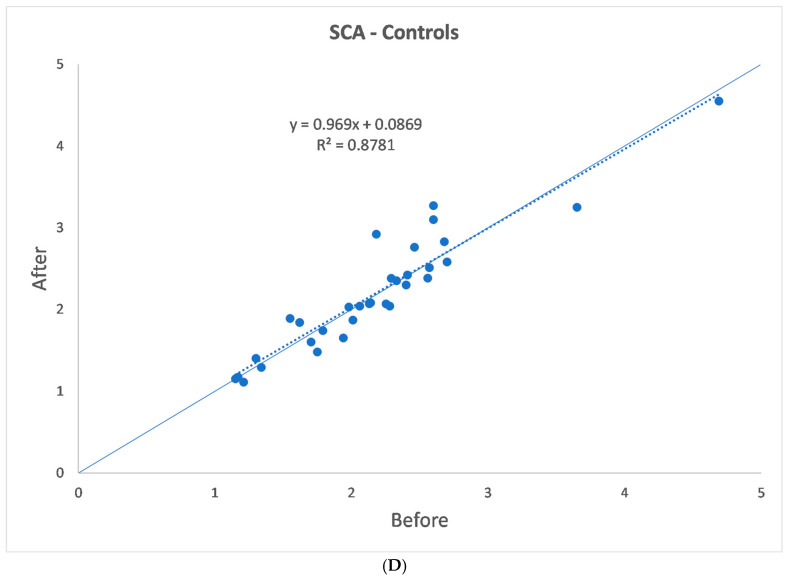
(**A**–**D**) Healthy controls’ choroidal vascularization parameters.

The SFCT, TCA, LCA, SCA, and CVI data are shown in Table 1(a,b) and Figure 2A–D and Figure 3A–D. One month after alfa-lytic therapy withdrawal, SFCT and LCA were significantly reduced (*p* = 0.007 and *p* = 0.045, respectively), whereas no significant difference in TCA, SCA, and CVI was found (*p* = 0.181, *p* = 0.806, and *p* = 0.248, respectively). In the control group, SFCT, TCA, LCA, SCA, and CVI measured at 1 month of distance showed no significant differences for all the evaluated parameters (*p* = 0.875, *p* = 0.654, *p* = 0.359, *p* = 0.424, and *p* = 0.545, respectively).

## 4. Discussion

Selective α1ARA inhibitors are the most common treatment in case of BPH and hypertension, and it is well known that the risk of IFIS during cataract surgery is increased in patients taking this kind of drug.

To avoid IFIS, Chang et al. [1] suggested α1ARA inhibitor discontinuation 1–2 weeks before surgery; however, the benefit and the time of discontinuation before surgery has not yet been established. A case report showed that a patient had IFIS during cataract surgery 7 weeks after taking a single dose of tamsulosin; therefore, the safe time to treat it is not clear and no predictive parameter to avoid IFIS is known [7].

In the past few years, several authors studied the α1ARA inhibitors’ effects to avoid IFIS and to discover predictive parameters [34,35,36,37].

In 74 patients treated with silodosin for six months compared to 30 healthy subjects, Karaca et al. [34] found higher values of amplitude and velocity of pupil contraction and lower values of both pupil contraction duration and latency, and duration and velocity of pupil dilation [34].

Safir et al. [35] found pupil diameter to be significantly lower among patients who developed IFIS compared with those who did not. Moreover, their results showed shallower ACD and larger lens thickness (LT) in the IFIS group [35].

Vural et al. [36] evaluated pupil size with infrared pupillometry under photopic (40 lx), mesopic (4 lx), and scotopic (0.04 lx) conditions, and found no statistically significant difference between treated and non-treated patients [36].

Casuccio et al. [37] examined the pupil size of patients taking tamsulosin for at least 1 year, patients taking another a1-ARA for at least 1 year, and control patients taking no a1-ARA. Pupil measurements were obtained using a computerized infrared pupillometer one month before surgery, under mesopic low (0.4 lx) and mesopic high (4.0 lx) illumination, and after mydriatic dilatation under mesopic high (4.0 lx) conditions. Preoperatively mydriasis was less in the tamsulosin group than in the other a1-ARA group, which was less than in the no-a1-ARA group.

Postoperatively, there were significant differences in pupil size between the three groups; the pupil size was smaller in the tamsulosin and in the other a1-ARA group than in the no-a1-ARA group. No difference was found between the tamsulosin group and the other a1-ARA group [37].

Despite the efforts of the authors, no conclusion results were obtained; in fact, three articles proved a correlation between pupil size and the risk of IFIS, whereas one article did not. Only one article showed a correlation between preoperative ACD and LT with risk of IFIS.

In the attempt to find a method that could be able to predict the risk of IFIS, two papers evaluated the presence of eventual choroidal changes. Unfortunately, they showed different results [6,7].

In the first study, Sari et al. [6] utilized an EDI-OCT (ZEISS Cirrus HDOCT 4000) in 29 eyes of 29 patients with new BPH diagnosis before and 3 months after tamsulosin intake, and attempted to check for any drug-induced choroidal change. ChT at the subfoveal level and at 750 μm nasally and temporally from the fovea was measured. All three observed points showed a significant ChT increase, and a vasomotor effect was suggested [6].

Dogan et al. [7] evaluated 63 right eyes of 63 patients diagnosed with BPH (32 taking alfuzosin and 31 tamsulosin) with EDI-OCT (ZEISS Cirrus HD-OCT 4000). The measurements were performed at the subfoveal level and at 3 mm nasally and temporally from the fovea. Contrary to the first study, a statistically significant choroidal thickening was only observed in patients treated with alfuzosin and not with tamsulosin [7].

De Bernardo et al. [13] were the first to look for changes after alpha lytic-withdrawal. They found a decrease in both SFCT and ChT, 1.5 mm nasally and temporally from the fovea, and suggested these findings as a marker to look for in the attempt to avoid IFIS, as the decrease in ChT could be related to a decrease in the effect of such medications on the uvea [13].

EDI-OCT, introduced in 2008, allows precise in vivo ChT measurements and represents the most used method for choroidal imaging evaluation [20]. The OCT software shifts the zero-delay line below, rather than above, obtaining inverted images. Therefore, the operator captures EDI-OCT images more easily because the image is direct, rather than inverted. However, ChT measurements are not automatic, so the operators manually draw the line perpendicular to RPE to measure it and obtain comparable data. This methodology may allow the operators to distinguish the stromal from the luminal choroidal structure.

Utilizing the EDI-OCT foveal scan, Sonoda et al. [30] firstly described an automatic technique to assess choroidal vascularization through an image binarization process using the free software ImageJ. The OCT image was opened in ImageJ to establish LCA and SCA from TCA and to calculate CVI [30].

To the best of our knowledge, this is the first and only paper where not only SFCT but also TCA, LCA, SCA, and CVI have been evaluated after α1ARA inhibitor withdrawal.

This study has several limitations. First, the size of the sample is not very large; however, we used G*Power3.1 software 7 to assess the right sample size to avoid bias [33].

Second, although many papers proved no correlation between sex and ChT, the different number of males and females of the groups could be a bias. Unfortunately, alpha-lytic therapy is especially widespread in the male population, and therefore matching samples without sex differences is very difficult.

All statistical tests and the assessment of the power of the test were run utilizing a *p*-value of 5%; however, the decrease in LCA in patients that discontinued α1ARA inhibitors produced a significance of 0.045, and therefore a false positive rate of 4.5%.

Perhaps, if the significance of the tests had been set at a *p*-value of 0.01, LCA changes would not have been significant, and this could be considered a limitation.

In future studies, it would be interesting to investigate the influence of the period intake of α1ARA inhibitors on choroidal parameters

This study has several strengths. First, we had strict exclusion criteria, and we used only high-quality images for analysis.

Second, only one operator took the SFCT measurements and another operator analyzed choroidal parameters with ImageJ 1.52q software, to avoid the risk of obtaining bias of measure technique.

Third, all data were analyzed by a different operator with SPSS Software to avoid the risk of bias.

This study showed a significative reduction in SFCT and LCA, whereas TCA, SCA, and CVI were not modified in patients who discontinued alpha-lytic therapy.

The decrease in SFCT and LCA supports the results of a previous paper which reported an increase in SFCT with α1ARA inhibitor intake [13] but, more notably, the decrease in LCA suggests that most of the increase with the α1ARA inhibitor intake is due to a choroidal vasodilation and not to stromal changes.

If confirmed, this finding could be very important in IFIS management because, if the alpha-lytic therapy does not affect the choroidal stroma, probably the same happens in the iris [38,39].

In support of this idea, another paper examined 51 cadaveric eyes of 27 patients (14 with a history of tamsulosin use and 13 control patients) and highlighted that vessel coiling during pupillary dilation may be impaired with α1ARA down-regulation, and this loss of dynamic function could contribute to iris floppiness. Moreover, the authors showed no difference in iris stromal thickness [40].

In conclusion, after the discontinuation of α1ARA inhibitors, considering the non-significant changes in TCA, there was no statistically significant CVI change, despite a significant LCA decrease. These unexpected results could be explained by the absence of brightness standardization in CVI measurements. In fact, regardless of the attempt to adjust brightness, to date, a standardized brightness level has not been established [41].

Further studies in a larger number of patients are required to determine the amount of changes needed to avoid IFIS. In addition, to perform an OCT exam before α1ARA inhibitor administration in all patients would be helpful to establish the values that must be obtained with drug withdrawal before cataract surgery.

## Figures and Tables

**Table 1:** (**a**,**b**) Choroidal vascularization parameters before and after alpha-lytic suspension in patients and healthy controls, respectively. SFCT: Subfoveal choroidal thickness; TCA: Total choroidal area; LCA: Luminal choroidal area; SCA: Stromal choroidal area; CVI: Choroidal vascularity index.

**(a)**					
	**Before**				
	**SFCT**	**TCA**	**LCA**	**SCA**	**CVI**
**Mean**	213.84 µm	6.49 mm^2^	4.16 mm^2^	2.35 mm^2^	63.92%
**SD**	79.65 µm	2.34 mm^2^	1.51 mm^2^	0.88 mm^2^	2.77%
**Median**	220.00 µm	6.25 mm^2^	4.10 mm^2^	2.25 mm^2^	64.00%
**Min**	62.00 µm	2.50 mm^2^	1.50 mm^2^	0.80 mm^2^	59.80%
**Max**	387.00 µm	11.10 mm^2^	7.20 mm^2^	4.30 mm^2^	69.50%
	**After**				
	**SFCT**	**TCA**	**LCA**	**SCA**	**CVI**
**Mean**	205.91 µm	6.39 mm^2^	4.04 mm^2^	2.34 mm^2^	63.47%
**SD**	80.24 µm	2.19 mm^2^	1.37 mm^2^	0.87 mm^2^	2.55%
**Median**	214.50 µm	6.20 mm^2^	4.00 mm^2^	2.15 mm^2^	63.35%
**Min**	53.00 µm	2.40 mm^2^	1.70 mm^2^	0.70 mm^2^	57.30%
**Max**	392.00 µm	11.20 mm^2^	7.00 mm^2^	4.20 mm^2^	69.80%
***p***	**0.007**	0.181	**0.045**	0.806	0.248
**(b)**					
	**Before**				
	**SFCT**	**TCA**	**LCA**	**SCA**	**CVI**
**Mean**	187.94 µm	6.09 mm^2^	3.92 mm^2^	2.18 mm^2^	64.23%
**SD**	57.10 µm	2.02 mm^2^	1.33 mm^2^	0.71 mm^2^	1.97%
**Median**	196.50 µm	5.80 mm^2^	3.75 mm^2^	2.15 mm^2^	64.70%
**Min**	62.00 µm	3.30 mm^2^	2.10 mm^2^	1.20 mm^2^	59.50%
**Max**	298.00 µm	12.70 mm^2^	8.00 mm^2^	4.70 mm^2^	69.00%
	**After**				
	**SFCT**	**TCA**	**LCA**	**SCA**	**CVI**
**Mean**	188.00 µm	6.06 mm^2^	3.87 mm^2^	2.20 mm^2^	63.94%
**SD**	57.50 µm	1.92 mm^2^	1.23 mm^2^	0.74 mm^2^	3.27%
**Median**	196.50 µm	6.00 mm^2^	3.90 mm^2^	2.10 mm^2^	64.00%
**Min**	63.00 µm	3.10 mm^2^	2.00 mm^2^	1.10 mm^2^	56.30%
**Max**	299.00 µm	12.30 mm^2^	7.70 mm^2^	4.60 mm^2^	71.50%
***p***	0.875	0.654	0.359	0.424	0.545

## Data Availability

No new data were created.
